# Nutmeg Poisoning

**Published:** 1908-07-18

**Authors:** 


### Toxicology.

### NUTMEG POISONING.

The question of poisoning by nutmegs has been brought to the notice of the profession by Professor Cushny, in a paper read before the Royal Society of Medicine; and the subject is of much interest in that, although nutmegs and preparations from them are seldom prescribed medicinally in any quantity, they are in use in almost every household, and it is perhaps surprising that they are not taken in poisonous quantities more often than they are. Dr. G. B. Wallace has collected twenty-five cases of nutmeg poisoning from the literature of recent years, and it is probable that many other cases have never been recorded. The condition, therefore, is less uncommon than might be thought.

That nutmegs contain a soporific principle has long been recognised, and it is on this account, as well as by reason of the pleasant flavouring, that a little nutmeg has been long advocated as an ingredient of the night-cap glass of grog. Like so many other drugs, however, that which is soporific in some cases, and with certain doses, may be a deliriant in other cases or in different doses, and it was recorded by Lobelius, as long ago as 1576, that a woman might be made delirious should she indulge too freely in the nutmeg.

The recorded cases show that the toxic symptoms supervene in many instances from the ingestion of no more than one or one-and-a-half nutmegs at a time. One man was poisoned by a teaspoonful of the mace. Doubtless there are great individual differences in different persons in respect to the quantities of nutmeg that can be taken with impunity; but there can be no doubt that certain people have the idiosyncrasy to be materially upset by the ingestion of less than two nutmegs.

The symptoms consist of drowsiness passing into stupor, coming on in from one to six hours; the patient may have diplopia, as in acute alcoholism, and although he may be able to answer questions at first, the stupor often deepens until it is with difficulty that he can be aroused at all. Delirium is frequently present before the stupor stage, and there may be inability to recognise surroundings or persons. Spasmodic movements of the jaw have been recorded in some cases, whilst in others there has been continual idiotic laughter, or persistent disconnected talk. Giddiness is sometimes one of the earlier symptoms. Gastro-intestinal troubles are usually slight, though there may be a sense of burning in the epigastrium. The pupils are generally widely dilated, and dryness of the throat may be complained of, so that belladonna poisoning may be simulated. Cardiac symptoms are not often pronounced, but when they are present they may be very alarming, the pulse becoming small and thready, the feet cold, the face pale, the general condition being one of acute collapse.

The stupor lasts for something between four hours and two days, passing off gradually; complete recovery almost always results, although in one case, that of a boy of eight who took two nutmegs, death resulted in twenty hours. The symptoms present features which remind one of poisoning by opium, by belladonna, and by cannabis indica. The mixed condition of depression and stimulation is very striking, and the chief effect is upon the cerebral centres rather than upon the viscera with which the swallowed nutmeg itself comes into contact.

It has been proved experimentally that if the volatile oil, of which there is from 3 to 8 per cent. in different nutmegs, is first distilled off, no toxic effects are produced by the residue. The poisonous properties of the spice seem, therefore, to be contained in the essential oil.

Seeing that recovery is the rule, treatment need not be too drastic. The administration of an emetic would be wise, in order to remove any nutmeg that may remain undigested in the stomach; for the rest, the usual treatment of the collapsed state by hot blankets and hot bottles, by the administration of stimulants, and by the use of liquid food, is all that is usually required.

